# Remote Sensing Assessment of Safety Risk of Iron Tailings Pond Based on Runoff Coefficient

**DOI:** 10.3390/s18124373

**Published:** 2018-12-11

**Authors:** Defu Che, Aiman Liang, Xuexin Li, Baodong Ma

**Affiliations:** 1 Key Laboratory of Ministry of Education on Safe Mining of Deep Metal Mines, Northeastern University, Shenyang 110819, China; chedefu@mail.neu.edu.cn; 2 Institute for Geoinformatics & Digital Mine Research, Northeastern University, Shenyang 110819, China; liulingxi19950307@gmail.com (A.L.); lnslxx8686@gmail.com (X.L.)

**Keywords:** iron tailings pond, remote sensing, runoff coefficient, catchment area, risk assessment

## Abstract

Iron tailings ponds are engineered dam and dyke systems used to capture iron tailings. They are high-risk hazards with high potential energy. If the tailings dam broke, it would pose a serious threat to the surrounding ecological environment, residents’ lives, and property. Rainfall is one of the most important influencing factors causing the tailings dam break. This paper took Chengde Area, a typical iron-producing area, as the study area, and proposed a remote sensing method to evaluate the safety risk of tailings ponds under rainfall condition by using runoff coefficient and catchment area. Firstly, the vegetation coverage in the study area was estimated using the pixel dichotomy model, and the vegetation type was classified by the support vector machine (SVM) method from Landsat 8 OLI image. Based on DEM, the slope of the study area was extracted, and the catchment area of the tailings pond was plotted. Then, taking slope, vegetation coverage, and vegetation type as three influencing factors, the runoff coefficient was constructed by weight assignment of each factor using analytic hierarchy process (AHP) model in both quantitative and qualitative way. Finally, the safety risk of tailings ponds was assessed according to average runoff coefficient and catchment area in the study area. The results showed that there were 124 low-risk tailings ponds, 16 moderate-risk tailings ponds, and 4 high-risk tailings ponds in the study area. This method could be useful for selecting targeted tailings ponds for focused safety monitoring. Necessary monitoring measurements should be carried out for the high-risk and moderate-risk tailings ponds in rainy season.

## 1. Introduction

Mineral resources are one of the most important resources for human survival, which also bring a series of safety problems while they are exploited and utilized [1]. As an important part of beneficiation production, tailings pond is composed of one or more tailings dams stacking to intercept the valley mouth or enclosure. Tailings pond is used to store the solid waste left in the process of mining, which contains a small number of useful components and is no longer suitable for further separation under current economic and technological conditions. At present, the global demand for mineral products is still huge. After high-grade and accessible ore bodies are gradually exhausted, the mining industry will mainly extract low-grade ore bodies in the future [2]. Thus, it can be predicted that the tailings will continue to increase. Because it usually contains lots of harmful elements, rivers and farmland around the tailings pond may be polluted seriously through runoff and infiltration [3]. If tailings dam breaks, tailings and waste water will pour down, and the residential areas downstream will be destroyed seriously. Therefore, safety risk assessment of tailings pond is particularly important for disaster prevention [4].

In China, there are more than 12,000 tailings ponds, including 4910 dangerous and disease ponds. For instance, ultra-low-grade magnetite has been exploited since the beginning of the new century in China. Because of low concentration ratio, a large number of tailings would be produced when iron concentrate was generated [5]. As a result, there are a large number of tailings ponds in ultra-low-grade magnetite mining areas. Safety monitoring of tailings pond could grasp the safety situation of tailings pond timely, prevent the occurrence of tailings pond accidents, and reduce the risk level of tailings pond effectively.

At present, safety monitoring of tailings ponds mainly focuses on stability of tailings dam. For example, a hierarchical structure model was established according to the factors affecting the safe operation of the tailings pond dam, and a judgment matrix of the related factors was constructed by using Delphi method to determine the weight of each influencing factor [6]. GPS means was used to monitor the displacement of tailings dam and other indicators online [7]. However, the evaluation and analysis are mostly based on the indicator system of ground investigation or monitoring. There are two disadvantages to this kind of approach. On the one hand, ground investigation and monitoring are costly, inefficient, and coarse. Tailings ponds are often located in poor conditions with inconvenient traffic, resulting in relatively high cost and low efficiency of field investigation. Furthermore, the result accuracy is usually coarse because a lot of index information (e.g., distance information) is usually determined through inquiry or visual estimation. On the other hand, the selection of indicators is partial to microscopic, but lacks support of macroscopic background. Presently, most of the monitoring and assessing indicators are related to the tailings pond itself, such as the displacement of some discrete points on the dam, and the change of the immersion line [8,9]. On the contrary, the indicators about the regional and macroscopic background are not considered fully. Flood is one of the main factors causing tailings dam break [10], which is related to runoff coefficient in the regional and macroscopic background of tailings ponds [11].

Runoff coefficient can indicate how much water becomes runoff in a given precipitation, and it can reflect the impact of natural geographical elements on runoff comprehensively [12]. Several factors, such as rainfall intensity, soil type, terrain, and land use, would influence the runoff coefficient [13,14,15]. The runoff coefficient is usually determined by the observed data of rainfall and runoff in a rational manner [16]. Unfortunately, internal variabilities are not taken into consideration in the approach because the observation is often implemented in large-scale areas [17]. Kennessey method is another typical deterministic method to calculate runoff coefficient [18]. Specific physiographic characteristics and climatic parameters are both used in this method. However, it is difficult to obtain all the accurate value of the physiographic and climatic parameters in practice. This deficiency could be made up by the analytic hierarchy process (AHP) method, which could consider both qualitative and quantitative factors [19]. AHP is a multi-criteria decision making method helping decision-makers facing a complex problem with multiple conflicting and subjective criteria [20]. It is widely used in the risk assessment [21,22]. Determining the influencing factors of decision goal is the basic step of AHP method. Tailings ponds are mostly located in valleys. In these areas, land surface is usually covered by vegetation under different slope. In addition, the land use and soil type would not change in a small-scale mountainous area. Therefore, only slope and vegetation information would be considered for calculating runoff coefficient around tailings ponds. Remote sensing can provide timely, accurate monitoring results by retrieving the slope and vegetation information of land surface pixel by pixel [23,24,25,26]. Thus, remote sensing could be used to provide the input parameters for the AHP model of runoff coefficient around tailings ponds.

The objective of this paper is to present a method towards assessing the safety risk of tailings ponds by estimating the runoff coefficient in the catchment area of tailings ponds by using AHP model. In this study, slope, vegetation coverage and vegetation type were considered and retrieved from remote sensing data.

## 2. Materials and Methods

### 2.1. Study Area

**Figure 1:**
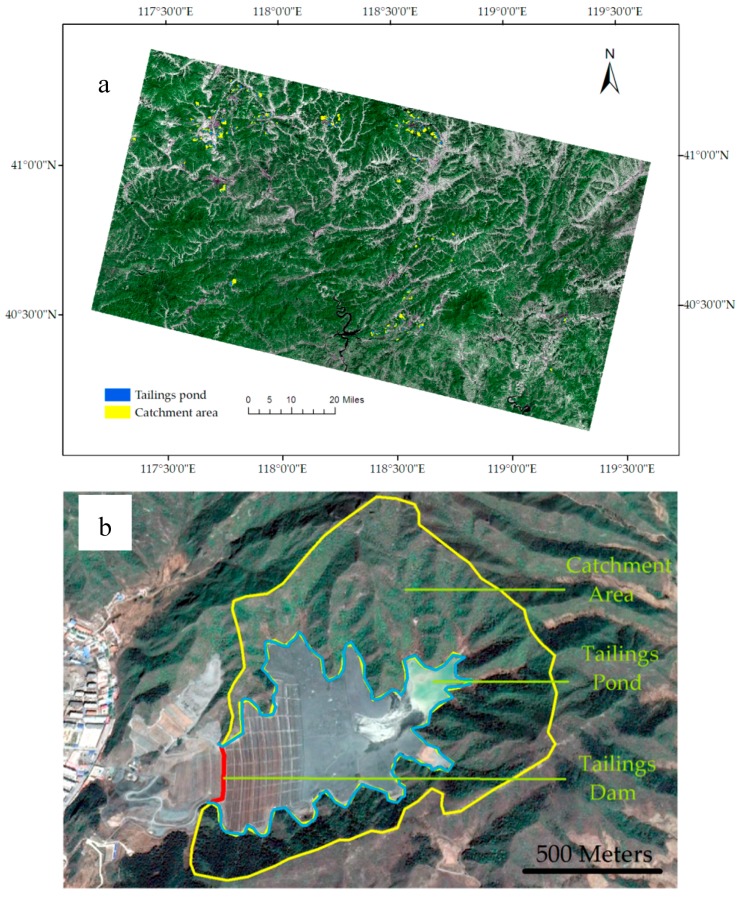
The study area and a typical iron tailings pond. (**a**) The study area (Landsat 8 OLI image); (**b**) A typical iron tailings pond in the study area (GoogleEarth image).

The study area is located in the southeast of Chengde Area, Hebei Province, North China. It is mountainous area with an elevation from 400 m to 900 m. The soil type is brown soil in this area. There are four major river systems in this area, including Chaohe river, Dalinghe river, Luanhe river and Liaohe river, with an annual water capacity of 3.76 billion cubic meters. More than 3.014 billion tons of ultra-low-grade magnetite ore were found, and more than 140 iron tailings ponds were produced in this area (Figure 1a). These tailings ponds were built at mouth of valley respectively (Figure 1b). Therefore, the tailings dams were under the threat of flood in the valley. In the whole study area, the main land-use types are farmland, residential area, woodland, and grassland. However, the land-use types are only woodland and grassland in the catchment area of tailings ponds.

### 2.2. Data Used

Landsat 8 OLI data were selected as remote sensing images to retrieve vegetation information. They were acquired on 15 August 2015, with a spatial resolution of 30 m, a track number of 122/32, and a projected coordinate system of UTM-WGS84 (http://www. dsac.cn/). By using ENVI software, the original image was calibrated by atmospheric and radiometric correction, and the digital number (DN) of the image was converted to surface reflectance.

Digital Elevation Model (DEM) data were with a spatial resolution of 30 m, and a projected coordinate system of UTM-WGS84 (http://www.dsac.cn/). DEM data were selected to retrieve the slope and catchment area in the study. The study area was covered with 12 DEM images, which were mosaic by referencing to Landsat 8 OLI image in the study area.

### 2.3. Retrieval of Influencing Factors of Runoff Coefficient Using Remote Sensing

**Figure 2:**
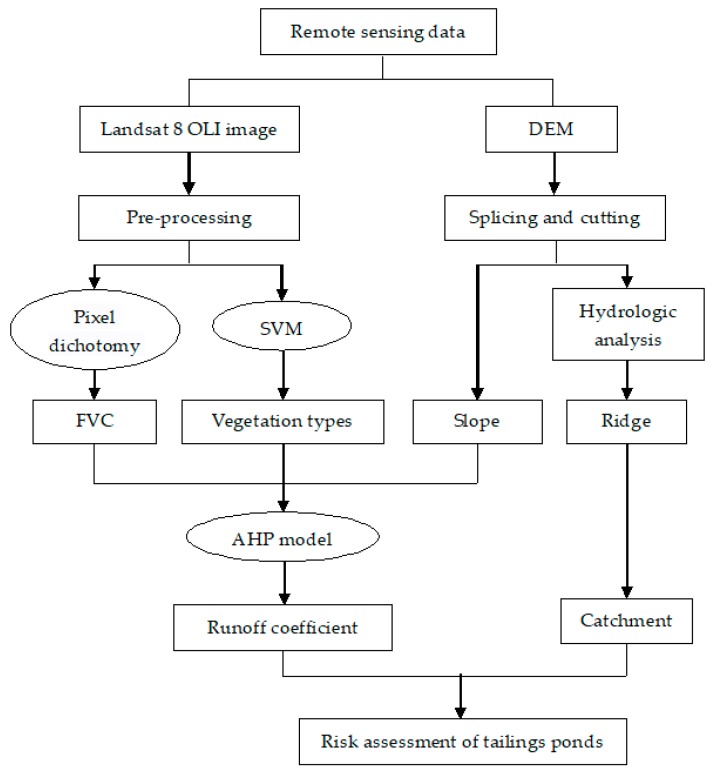
Flow chart of risk assessment for tailings ponds.

Due to the differences of actual geographical environment in each region and the frequent occurrence of extreme weather in recent years, the runoff coefficient in different regions can’t be identified by a single factor. In the study area with the same rainfall condition and soil type, the underlying surface slope has direct influence on the change of surface runoff generation, and the runoff coefficient would increase gradually with the increase of slope. In other words, slope is one of the main factors affecting runoff generation [27]. Furthermore, the hydrological effects of plants have a certain influence on the runoff coefficient. Concretely, different- vegetation type and coverage have different functionality of rainfall interception and blockage runoff [28]. Therefore, the three factors, slope, vegetation coverage and vegetation type, were selected to determine the runoff coefficient of the area using AHP model. The safety risk of the tailings pond was assessed according to the catchment area and average runoff coefficient of each tailings pond. The flowchart is shown in Figure 2.

#### 2.3.1. Fractional Vegetation Coverage (FVC)

Vegetation plays an important role in soil consolidation and storm elimination [29]. Fractional Vegetation Coverage (FVC) is an important parameter reflecting the distribution of surface vegetation and an indicator reflecting the surface ecological environment [30]. FVC of the radiation range of the mining area is closely related to the diggings exploitation situation and the type of ore. With increase of coverage, the interception effect of vegetation on rainfall is strengthened. At the same time, the infiltration performance of soil is improved, which can effectively intercept and store the peak discharge, reduce runoff coefficient, and reduce the occurrence probability of geological disasters [31].

Many methods have been developed to retrieve FVC by remote sensing. The widely used method is to estimate fractional vegetation coverage by NDVI and pixel dichotomy model [32]. The pixel dichotomy model is a simple and practical remote sensing estimation model. It assumes that a pixel is consists of vegetated area and non-vegetated area. The spectral information observed by the remote sensing sensor is also linearly weighted by the component. The pixel dichotomy model is as follows:

(1) FVC=(NDVI−NDVImin)(NDVImax−NDVImin)

where NDVI_max_ and NDVI_min_ are the maximum and minimum NDVI values in the region. Due to the inevitable noise, NDVI_max_, and NDVI_min_ would take the maximum and minimum values within a certain confidence level range. The value of confidence level is mainly determined according to the actual situation of the image. In this paper, according to the statistical results of the study area, NDVI values with accumulative probability of 5% and 95% were respectively taken as NDVI_min_ and NDVI_max_.

#### 2.3.2. Vegetation Types

The amount of rainfall retaining by vegetation is different from the vegetation types [33]. In this paper, support vector machine (SVM) was selected as the classifier for vegetation classification. SVM is a machine learning method based on statistical learning theory. It applies the principle of structural risk minimization to the classification field [34]. In this study area, there are two main vegetation types, grass, and woods. Therefore, the sample categories of SVM classifier include grassland, woodland, and others (e.g., bare land, building area).

#### 2.3.3. Slope

The location of the iron tailings ponds in this area is in the valley. The surrounding terrain would threat the safety of tailings pond. With the increase of slope, the runoff coefficient would become higher and higher [35]. Therefore, excessive slope may lead to flood easily in rainy weather because of the high runoff. Thus, slope is an important factor affecting the safety of tailings ponds [36].

DEM data can derive landform characteristics, including slope gradient, slope aspect, shadow landform map, and surface curvature. It could be used to calculate the terrain feature parameters, including mountain peaks, ridges, river courses, and gullies. By using ENVI software and DEM data of the study area, the size of topographic core was set to 5, multi-scale topographic information was extracted, and the slope value of each pixel was extracted.

### 2.4. Calculation of Runoff Coefficient Based on AHP Model

In this paper, the risk assessment of tailings pond was based on the weight of accidents caused by above impact factors. There are a number of analytical models for determining the weight of each factor, such as experimental analogy, mathematical modeling, forced pair comparison, AHP and so on [37]. Among them, AHP is the commonly used model. It is a multiple-dimensional decision-making method that combines qualitative and quantitative methods. It uses mathematical methods to calculate the weights that reflect the relative importance of the elements at each level. Then it calculates the relative weights of all elements by the total ordering between all levels and sorts them [38]. Firstly, it is necessary to establish a hierarchical model, and identify the objectives of the decision, the factors considered (decision criteria) and the decision object. Then, it needs to construct a judgment matrix according to the influence degree of each factor of a certain layer on a certain factor of the upper layer. Finally, the hierarchical ordering and the consistency test are carried out, that is, the logical consistency of the thinking is judged to avoid the conflicting judgments. According to the judgment matrix, the eigenvector corresponding to the largest characteristic root is obtained. The eigenvectors obtained are the order of importance of each evaluation factor.

**Figure 3:**
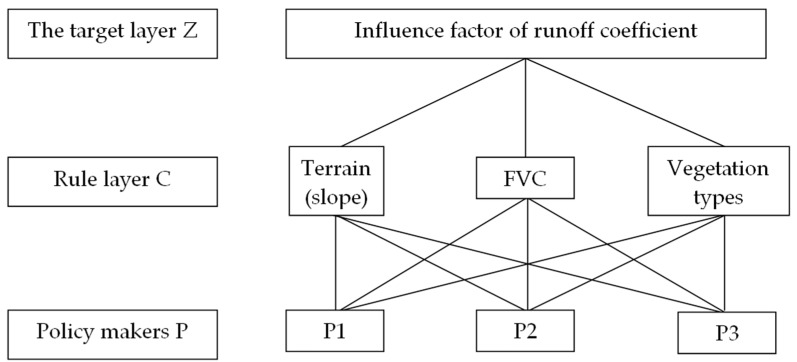
Diagram of Analytic Hierarchy Process for the influencing factors of runoff coefficient.

When constructing a judgment matrix, it is necessary to compare the relative importance of different factors in the rule layer. Terrain plays a more important role than vegetation coverage in affecting the runoff coefficient [39,40]. The vegetation’s function of water conservation can increase the infiltration time of runoff by increasing the surface roughness in the ground part and increase the infiltration of runoff by improving the physical and chemical properties of soil in the ground part. Therefore, the effect of vegetation coverage on runoff coefficient is greater than that of vegetation type [18]. On this basis, the relative importance of three factors was determined, and the three factors were compared in pairs, and the judgment matrix of runoff coefficient was constructed. At last, three kinds of solutions, namely P1, P2, and P3, were obtained (Figure 3).

### 2.5. Extraction of Catchment Area of Tailings Pond and Risk Assessing

Besides runoff coefficient, size of catchment area also plays an important role in determining surface runoff [41]. The catchment area was drawn along the ridge line. By extracting the grid of zero sink flow accumulation, the ridge line can be obtained [42]. Particularly, the catchment area of each tailings pond is the upper part of the whole catchment of the valley, which is divided by the tailings dam. Then, average runoff coefficient in each catchment area was calculated respectively. Both catchment area and its average runoff coefficient are the determining factors for the risk assessment of each tailings pond. Therefore, the product result of runoff coefficient and catchment area, namely risk index, was taken to assess the risk of tailings ponds.

## 3. Results

### 3.1. Runoff Coefficient Calculated Based on Three Factors

**Figure 4:**
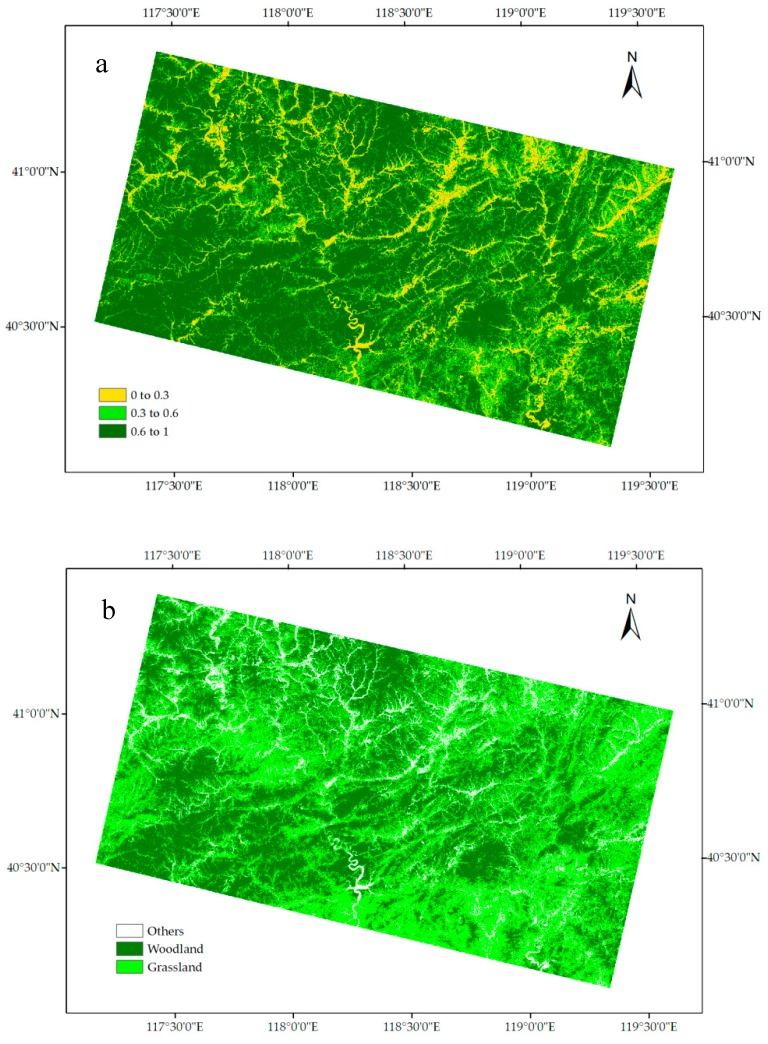

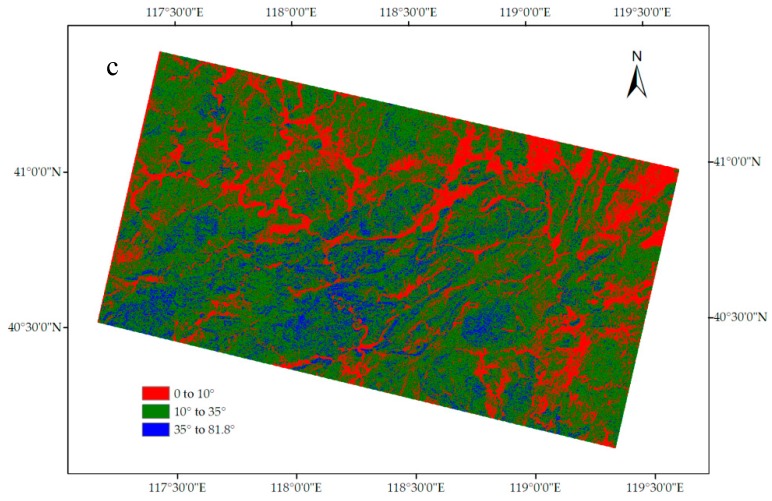
Remote sensing retrieval of vegetation coverage, vegetation type, and slope. (**a**) Vegetation coverage level map based on FVC results; (**b**) Vegetation types map in the study area (In this map, bare land and rivers are classified as “others”); (**c**) Slope level of the study area.

By using pixel dichotomy model, FVC in the study area was retrieved and divided into three levels (Figure 4a). In detail, pixels with FVC from 0 to 0.3 were classified as the first level, pixels with FVC from 0.3 to 0.6 were classified as the second level, and pixels with FVC from 0.6 to 1 were classified as the third level. By using SVM classifier, the image was classified into three classes in the study area (Figure 4b). In detail, grassland was set as the second level, and woodland was set as the third level, and others were set as the first level. Slope information was retrieved and then divided into three levels (Figure 4c). In detail, pixels with slope from 0 to 10 degrees were classified as the first level, pixels with slope from 10 to 35 degrees were classified as the second level, and pixels with slope more than 35 degrees were classified as the third level.

According to the relevant literature [18,43,44], the runoff coefficient corresponding to three influencing factors under different levels were obtained (Table 1).

**Figure 5:**
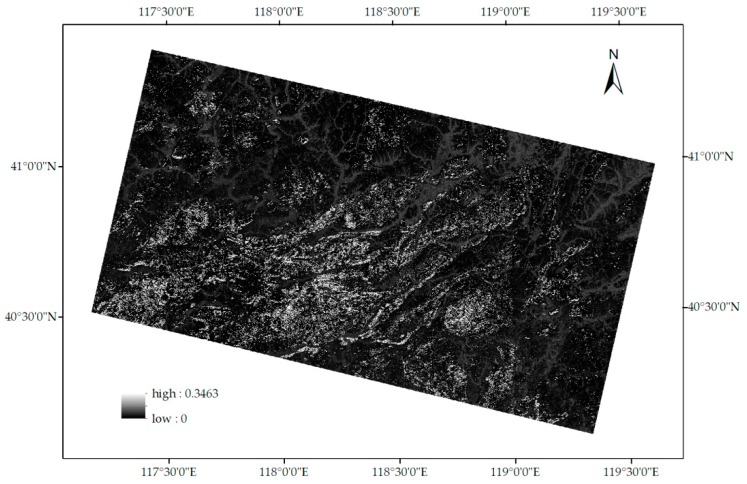
Runoff coefficients in the study area.

According to AHP model, the weight of slope, FVC, and vegetation type for determining the runoff coefficient was 0.74, 0.17, and 0.09 respectively. The mathematical calculation model of the runoff coefficient was established as follows:

(2) A=0.74S+0.17V+0.09T,

where A is the runoff coefficient of a pixel, S is the runoff coefficient corresponding to the pixel’s slope level, V is the runoff coefficient corresponding to the pixel’s FVC level, and T is the runoff coefficient corresponding to the pixel’s vegetation type. The runoff coefficients were obtained pixel by pixel (Figure 5).

### 3.2. Risk Analysis of Tailings Ponds

The catchment area of each tailings pond was extracted by using ridge line (Figure 1a). The risk index, product result of average runoff coefficient and catchment area, was calculated for assessing the safety risk each tailings pond (Table 2). The greater the risk index, the greater the probability of tailings dam-break accident caused by flood.

**Figure 6:**
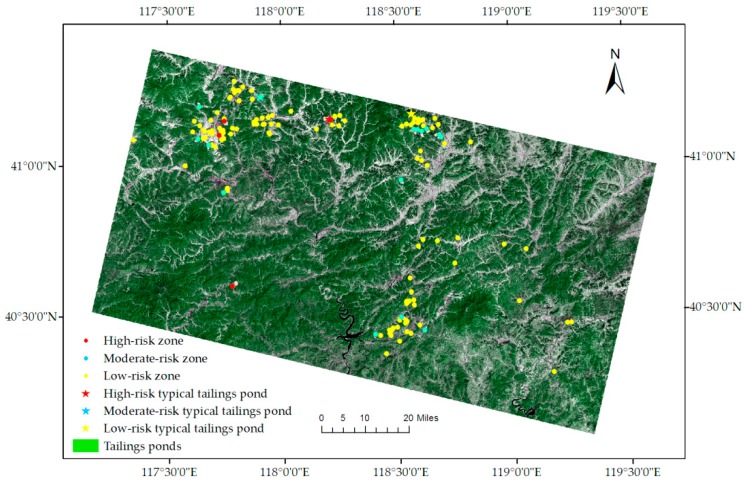
Risk assessment of all the tailings ponds in the study area (The five-star symbol means the selected example in Figure 7).

Risk index of each tailings pond was analyzed and risk grading was conducted for 144 tailings ponds in the study area. The overall risk index range was from 0.132 to 5.665. The tailings ponds with 0.132–1.920 risk index were set as the low-risk ponds, the ones with risk index of 1.920–3.500 were the moderate-risk ponds, and the ones with 3.500–5.665 risk index were the high-risk ponds. The results showed that, there were 124 low-risk, 16 moderate-risk, and 4 high-risk tailings ponds among the total 144 tailings ponds (Table 3, Figure 6).

## 4. Discussion

**Figure 7:**
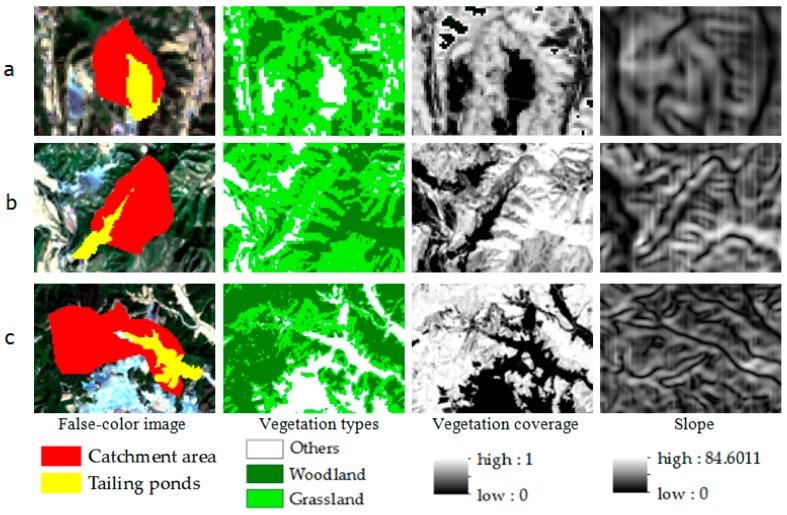
Three typical tailings ponds with different risk levels in catchment area, vegetation coverage, vegetation types and slope. (**a**) The low-risk tailings pond; (**b**) The moderate-risk tailings pond; (**c**) The high-risk tailings pond. The location of the three tailings ponds is labeled with five-star symbols in Figure 6.

The results of this study could provide the risk level of the threat of flood for these tailings ponds. According to the assessing results, a total of 124 tailings ponds are in low-risk condition. Around these tailings ponds, the slope of the terrain is gentle, the vegetation coverage is high, and the catchment area is small (Figure 7a). The 16 tailings ponds are in moderate-risk condition. It is necessary to strengthen supervision for these tailings ponds (Figure 7b). Four tailings ponds were assessed as high-risk tailings pond with steep topography slope, large catchment area, and relatively low vegetation coverage around them (Figure 7c). Once heavy rainfall occurs, there will be high risk of dam break for them. Therefore, it needs continuous monitoring, regular maintenance, management for these high-risk tailings ponds in rainy season.

However, scale effect was not considered in the study. Runoff coefficient is dependent on the scale [15,45]. In the study, the catchment area of tailings pond is small-scale area with a few square kilometers. Therefore, the initial runoff coefficients corresponding to three influencing factors in the AHP model may be not very suited for the small-scale catchment. Therefore, the field measurement should be carried out to measure the rainfall and runoff in the future around some typical tailings ponds. The field records could also be used to verify the assessing results.

The safety risk of the tailings ponds was assessed based on the runoff coefficients and catchment area in the study. The macroscopic background of tailings pond was considered fully. However, from a comprehensive perspective, characteristics of tailings dam and tailings pond could be considered along with above factors. For tailings dam, the structure and behavior condition are the main factors for the safety [46]. For tailings pond, it is important to understand the physical and chemical properties of stored tailings for the safety assessment [47]. Furthermore, potential slide and failure of these tailings ponds should be another important aspect for the comprehensive risk assessment [48]. Numerical modelling method applied in the landslides study offers important reference for the future study [49,50,51]. For example, geometry of landslides and their rotation with displacement could be simulated by using a multi-block sliding model, which was proved to be useful for predicting post-failure landslide displacement [52]. Therefore, the nature of the tailings and dam, the hydrological background in the catchment area, and the post-failure prediction would be studied comprehensively in our future study.

## 5. Conclusions

There are large number of tailings ponds in Chengde Area. Tailings ponds pose a threat to production safety and the lives of local residents. During the rainy season, the threat becomes greatest because these tailings ponds may be destroyed by the torrential flood. Manual investigation of the risk assessment would be very time-consuming and inefficient in the mountainous area. By using remote sensing methods, influencing factors of runoff coefficient, vegetation coverage, vegetation type, and slope, and catchment information could be retrieved timely. AHP model is useful for estimating runoff coefficient by weighing these factors. The risk assessment of tailings ponds considering runoff coefficient and catchment area would benefit the decision maker to monitor the targeted tailings ponds in rainy season.

According to the assessing result, it was found that there were 124 low-risk tailings ponds, 16 moderate-risk tailings ponds and 4 high-risk tailings ponds in the study area. In a word, more attention should be paid to the high-risk and moderate-risk tailings ponds in rainy season. On the one hand, the drainage system of tailings ponds must keep normal function. In case of heavy rainfall, the runoff would be drained timely by the drainage system and pose little threat to the dams. On the other hand, necessary monitoring equipment should be installed in the high-risk and moderate-risk tailings ponds, and monitoring staff should inspect them timely in rainy season. All in all, the assessment and corresponding measures could reduce safety risk of tailings ponds facing flood.

## Figures and Tables

**Table 1:** Runoff coefficient of different levels of each influencing factor.

Level	Slope	FVC	Vegetation Types
First level	0.02	0.16	0.27
Second level	0.14	0.08	0.19
Third level	0.24	0.04	0.04

**Table 2:** Calculation results of risk assessment indexes for some tailings pond.

Tailings Pond Number	Runoff Coefficient	Catchment Area/km^2^	Risk Index
1	0.156	9.360	1.460
2	0.147	5.184	0.762
3	0.120	5.598	0.672
4	0.142	4.086	0.580
5	0.129	12.744	1.644
…	…	…	…
142	0.141	7.263	1.024
143	0.118	2.133	0.252
144	0.163	5.985	0.976

**Table 3:** Risk assessment of tailings ponds.

Quantity of Tailings Ponds	Risk Assessment
124	Low-risk zone
16	Moderate-risk zone
4	High-risk zone
